# 12-Month clinical and radiographic outcomes of ViBone viable bone matrix in patients undergoing cervical and lumbar spinal fusion surgery

**DOI:** 10.1186/s13018-023-03686-9

**Published:** 2023-03-25

**Authors:** Paul D. Kim, Ramin Raiszadeh, David A. Bomback, David L. Kramer, Michael Moghimi

**Affiliations:** 1 Spine Institute of San Diego, 6719 Alvarado Road Suite 308, San Diego, CA 92120 USA; 2 Connecticut Neck and Back Specialists, 39 Hospital Ave, Danbury, CT 06810 USA; 3 Orthopaedic Specialists of Austin, 4611 Guadalupe Street Suite 200, Austin, TX 78751 USA

**Keywords:** Cervical spine, Lumbar spine, Spine fusion, Viable bone matrix, Bone allograft

## Abstract

**Background:**

To investigate the clinical safety and efficacy of ViBone^®^ Viable Bone Matrix (VBM), a next generation cellular bone matrix allograft that comprises all three essential bone-forming components: osteogenic, osteoinductive, and osteoconductive factors, and is optimized to enhance cell viability and bone formation.

**Methods:**

This was a multi-center, prospective, post-market study evaluating the safety and efficacy of ViBone VBM in patients undergoing 1–3 level anterior cervical discectomy and fusion or lumbar interbody fusion surgery. Patients were evaluated at baseline, 6-month, and 12-month follow-up clinically and radiographically. Clinical assessment included Visual Analog Scale for pain (VAS-pain), the Neck Disability Index (NDI) for patients with cervical pathologies, and the Oswestry Disability Index (ODI) for patients with lumbar pathologies. Fusion success defined by an independent radiologist was determined radiographically by plain films.

**Results:**

Clinical outcomes evaluated with VAS-pain, NDI, and ODI scales were improved significantly at 6 and 12 months compared to baseline. All patients reached clinically significant improvements at 12 months. There were no adverse events or infections attributed to ViBone VBM. At 12 months, the fusion rate per patient was 88.1% in cervical and 97.6% in lumbar patients, while per-level fusion was 98.5% for cervical and 100% for lumbar segments.

**Conclusions:**

Patients undergoing cervical and lumbar spinal fusion implanted with ViBone VBM demonstrated favorable outcomes at 6 months and 12 months as measured by subjective clinical measures and radiographic fusion rates.

*Trial registration* This study was registered as NCT03425682 on 1/29/2018.

## Background

Spinal fusion is commonly performed to treat various spinal conditions, and the rate of these procedures have been increasing faster than other orthopedic procedures: a 137% increase was reported in the annual number of spinal fusion discharges between 1998 and 2008 in the United States, whereas laminectomy, and hip and knee arthroplasty increased by 11.3%, 49.1%, and 126.8% respectfully [1, 2]. This significant increase in surgical volume led to the development and expansion of bone graft substitutes to improve patient outcomes.

The primary goal of spinal fusion therapy is to achieve solid bony arthrodesis [3]. Autologous iliac crest bone graft (ICBG) has been considered the gold standard for spinal fusion because it possesses all three necessary properties for new bone formation: osteogenic, osteoinductive, and osteoconductive factors [4]. However, harvest of ICBG may be variable in quantity and quality depending on patient factors and is associated with increased morbidities such as chronic harvest site pain (reported in up to 15% of patients), infection, iatrogenic fracture, and hematoma [3, 5, 6]. Bone graft substitutes provide a safe and effective alternative that avoids these potentially serious complications [7].

A variety of bone graft alternatives are commercially available; however, few substitutes contain all the essential components required for successful arthrodesis [8]. Human allograft bone graft substitutes, such as off-the-shelf viable bone matrix (VBM), are an attractive alternative to ICBG because they contain all three essential bone-forming components for fusion. A critical review concluded that VBMs may be superior to ICBG because of their high reported fusion rates and low complications [9].

ViBone VBM is a next generation human cellular allograft bone matrix comprised of the same bone-forming elements as ICBG and has been employed for bone repair in spinal, orthopedic, maxillofacial, and other various procedures since it was commercially available per the FDA as a human cells, tissues, and cellular and tissue-based product (HCT/P) in 2017. The unique features of this VBM are due to optimized manufacturing processes to improve handling characteristics, delivering cohesiveness and ease of molding for various implantation sites, and enhance osteogenic potential by preserving cellular health and minimizing apoptotic activity within the allograft. The aim of this study was to collect our real-world clinical experience with ViBone in various spinal fusion procedures.

## Methods

### Study population

This prospective, multicenter, post-market registry study collecting clinical and radiographic outcomes was conducted to evaluate the safety and efficacy of ViBone in patients 18–80 years of age with spondylosis, spondylolisthesis, degenerative disc disease, or herniated nucleus pulposus. Cervical fusion patients were included if they underwent anterior cervical discectomy and fusion (ACDF) surgery using ViBone at 1–3 contiguous levels between C2 and C7; included lumbar fusion patients underwent lumbar interbody fusion surgery using ViBone at 1 to 3 contiguous levels between L1 and S1. Each site obtained IRB approval prior to the initiation of enrollment, and patients were provided with written informed consent before participating in any study-related activities. Exclusion criteria were: patients on long-term medications known to inhibit fusion or bone metabolism, or immune suppressants 6 months before surgery; treatment with radiotherapy; cervical and lumbar fusion during the same procedure; acute/chronic systemic/localized spinal infections; instability associated with major reconstructive surgery for primary/metastatic malignant tumors of the cervical/lumbar spine; previous pseudoarthrosis at any level of the cervical/lumbar spine; current/recent history of malignancy or infectious disease; inability to provide informed consent; rapid joint disease, bone absorption, osteomalacia, and/or diagnosed osteoporosis (Z score of ≤ − 2.5); any other medical/surgical condition which would preclude the potential benefit of surgery. Safety evaluation included incidence of serious adverse events related to the use of ViBone.

### Primary endpoint

Fusion status per independent radiographic examination with plain films was the primary endpoint. Radiographic images were collected at baseline, 6 months, and 12 months post-operatively and evaluated by an independent radiologist using the Bridwell interbody fusion grading system ranging from Grade I to Grade IV (Table 1) [10]. Grade I indicates fusion with remodeling and trabeculae. In Grade II the graft is intact but not fully remodeled and incorporated. Grade III represents an intact graft but with associated lucency. Grade IV represents no fusion with resorption of bone graft. Grades I and II were defined as successfully fused, based on previous literature [11].

**Table 1:** Bridwell interbody fusion grading system

Grade I	Fused with remodeling and trabeculae present
Grade II	Graft intact, not fully remodeled and incorporated, but no lucency present
Grade III	Graft intact, potential lucency present at top and bottom of graft
Grade IV	Fusion absent with collapse/resorption of graft

### Secondary endpoints

Secondary endpoints included self-reported subjective evaluations for patient pain and function: Visual Analog Scale for pain (VAS-pain), Neck Disability Index (NDI) for patients with cervical pathologies, and Oswestry Disability Index (ODI) for patients with lumbar conditions. Our VAS-pain scale ranged from 0 to 10 with 0 indicating no pain and 10 indicating severe pain with activity. The NDI and ODI tests used ranged from 0 to a maximum raw score of 50 with scores in this study reported as percentages (high scores indicate greater disability). Clinically significant improvements were considered a decrease of 2.6 points (cervical) or 2 points (lumbar) for VAS-pain, − 17.3% points for NDI, and -10% points for ODI as established in the literature [12, 13].

### Statistical analysis

Analyses for this study were descriptive. Our cohort is described combined where appropriate and split by cervical and lumbar cases. Continuous variables are reported using means with standard deviations and categorical data are reported using counts with percentages. Pain and disability scores are reported as continuous; fusion rates as noncontinuous. Paired samples t-tests were used to evaluate change in scores from baseline to 6- and 12-months postoperative. Significance was set at *p* < 0.05. SPSS was used for statistical analysis (IBM Statistics for Windows, Version 27.0. Armonk, NY: IBM Corp).

## Results

### Patient and procedural information

A total of 104 patients were enrolled in the study from four centers. Nine (9) patients were excluded from statistical analysis for either lack of follow-up (n = 6), or patient self-withdrew from study (n = 3). Thus, 95 patients (48 cervical; 47 lumbar) were included in our data analysis because they met the inclusion criteria and had at least one follow-up (6- and/or 12-month). There were 48 (50.5%) male and 47 (49.5%) female patients with a mean age of 56.2 ± 9.4 years (range 32–78). Patient demographics and clinical characteristics are described in Table 2.

**Table 2:** Patient demographics and clinical characteristics

	All (n = 95)	Cervical (n = 48)	Lumbar (n = 47)
Age (years, Mean ± SD)	56.2 ± 9.4	54.6 ± 9.1	57.8 ± 9.5
*Gender*			
Male	48 (50.5%)	25 (52.1%)	23 (48.9%)
Female	47 (49.5%)	23 (47.9%)	24 (51.1%)
*Race*			
White	60 (63.2%)	25 (52.1%)	35 (74.5%)
Hispanic	25 (26.3%)	15 (31.3%)	10 (21.3%)
Black or African American	6 (6.3%)	4 (8.3%)	2 (4.3%)
Other	4 (4.2%)	4 (8.3%)	0 (0.0%)
BMI (Mean ± SD)	28.8 ± 5.5	27.9 ± 5.7	29.8 ± 5.2
*BMI-category*			
Normal (18.5–< 25.0)	21 (22.1%)	15 (31.3%)	6 (12.8%)
Overweight (25.0–< 30.0)	42 (44.2%)	17 (35.4%)	25 (53.2%)
Obese (30.0–< 40.0)	30 (31.6%)	16 (33.3%)	14 (29.8%)
Morbidly obese (40.0 +)	2 (2.1%)	0 (0.0%)	2 (4.3%)
*Smoking*			
Never	71 (74.7%)	34 (70.8%)	37 (78.7%)
Former	17 (17.9%)	8 (16.7%)	9 (19.1%)
Current	7 (7.4%)	6 (12.5%)	1 (2.1%)
*Relevant medical history*			
# per patient, (Mean ± SD)	1.0 ± 1.0	1.0 ± 0.9	1.0 ± 1.0
None	37 (38.9%)	18 (37.5%)	19 (40.4%)
Diabetes, Type 2	9 (9.5%)	9 (18.8%)	0 (0.0%)
Diabetes-unknown type	4 (4.2%)	0 (0.0%)	4 (8.5%)
Hypothyroidism/thyroid problems/issues	13 (13.7%)	6 (12.5%)	7 (14.9%)
AFIB	3 (3.2%)	1 (2.1%)	2 (4.3%)
CAD	2 (2.1%)	0 (0.0%)	2 (4.3%)
History of Cancer	7 (7.4%)	5 (10.4%)	2 (4.3%)
Hypertension	21 (22.1%)	10 (20.8%)	11 (23.4%)

There were no instances reported of Paget’s Disease (0.0%), or autoimmune disorders excluding rheumatoid arthritis (0.0%)

The most frequent diagnoses and indications for spinal fusion surgery were spinal stenosis (69.5%), Degenerative Disc Disease DDD (63.2%), spondylosis (51.6%), spondylolisthesis (26.3%), and herniated disc (14.7%) (Table 3). Most operations were performed using minimally invasive approaches (61.1%), and all procedures (100%) included interbody fusion and use of a spacer. Lumbar fusion surgical approaches are listed in Table 3.

**Table 3:** Procedural details

	All (n = 95)	Cervical (n = 48)	Lumbar (n = 47)
Indications for surgery (# per patient, Mean ± SD)	2.4 ± 0.9	2.3 ± 0.9	2.4 ± 1.0
Degenerative disc disease (DDD)	60 (63.2%)	34 (70.8%)	26 (55.3%)
Herniated nucleus pulposus (HNP)	14 (14.7%)	7 (14.6%)	7 (14.9%)
Spondylosis	49 (51.6%)	22 (45.8%)	27 (57.4%)
Spondylolisthesis	25 (26.3%)	1 (2.1%)	24 (51.1%)
Stenosis	66 (69.5%)	45 (93.8%)	21 (44.7%)
Other	9 (9.5%)	2 (4.2%)	7 (14.9%)
Surgery type			
ACDF	48 (50.5%)	48 (100.0%)	–
MIS	–	30 (62.5%)	–
Open	–	18 (37.5%)	–
ALIF	25 (26.3%)	–	25 (53.2%)
MIS	–	–	25 (100.0%)
TLIF	21 (22.1%)	–	21 (44.7%)
MIS	–	–	3 (14.3%)
Open	–	–	18 (85.7%)
PLIF	1 (1.1%)	–	1 (2.1%)
Open	–	–	1 (100.0%)
Levels treated	n = 160	n = 93	n = 67
# Levels Treated (1–3), (Mean ± SD)	1.7 ± 0.6	1.9 ± 0.6	1.4 ± 0.6
C3–C4	4 (2.5%)	4 (4.3%)	–
C4–C5	17 (10.6%)	17 (18.3%)	–
C5–C6	39 (24.4%)	39 (41.9%)	–
C6–C7	33 (20.6%)	33 (35.5%)	–
L2–L3	1 (0.6%)	–	1 (1.5%)
L3–L4	8 (5.0%)	–	8 (11.9%)
L4–L5	29 (18.1%)	–	29 (43.3%)
L5–S1	29 (18.1%)	–	29 (43.3%)
Revision of a previous surgery?	7 (7.4%)	2 (4.2%)	5 (10.6%)
Interbody implant used during procedure			
PEEK—Titanium Coating	34 (35.8%)	22 (45.8%)	12 (25.5%)
PEEK—Traditional	7 (7.4%)	0 (0.0%)	7 (14.9%)
Vertu stand-alone with Ti-Bond	21 (22.1%)	20 (41.7%)	1 (2.1%)
Titanium mesh cage	14 (14.7%)	0 (0.0%)	14 (29.8%)
Spinal elements magnum + with Ti-bond	11 (11.6%)	2 (4.2%)	9 (19.1%)
Spinal elements magnum + with mercury	2 (2.1%)	0 (0.0%)	2 (4.3%)
PEKK—Tetrafuse	2 (2.1%)	2 (4.2%)	0 (0.0%)
Other	4 (4.2%)	2 (4.2%)	2 (4.3%)
Total amount of ViBone used across all levels			
cc, (Mean ± SD)			
Volume used for 1 level	6.1 ± 3.0	2.8 ± 1.6	7.2 ± 2.6
Volume used for 2 levels	5.9 ± 3.0	4.0 ± 1.5	9.5 ± 1.4
Volume used for 3 levels	7.0 ± 4.6	4.7 ± 0.8	15.0 ± 0.0

The study protocol allowed up to 30% of other graft material to be used in addition to ViBone, per physician choice based on the patient’s clinical needs. Other graft materials used by study investigators were autologous local bone, platelet rich plasma, blood, bone marrow, cancellous bone chips, demineralized bone matrix (DBM), and other (non-DBM) allograft materials. No patients receiving ViBone required harvesting of ICBG.

### Subjective clinical outcomes

Pain and disability scores were collected at baseline, 6-month, and 12-month follow-up evaluations. Two lumbar patients did not present for their 6-month follow-up and a total of 23 patients (11 cervical; 12 lumbar) were lost to follow-up for various reasons at the 12-month visit—none of which were reported to have any adverse events or be deceased. Additionally, 6-month pain and disability scores were unavailable for two patients (1 cervical; 1 lumbar), and five patients at 12-month follow-up (1 cervical; 4 lumbar).

**Fig. 1:**
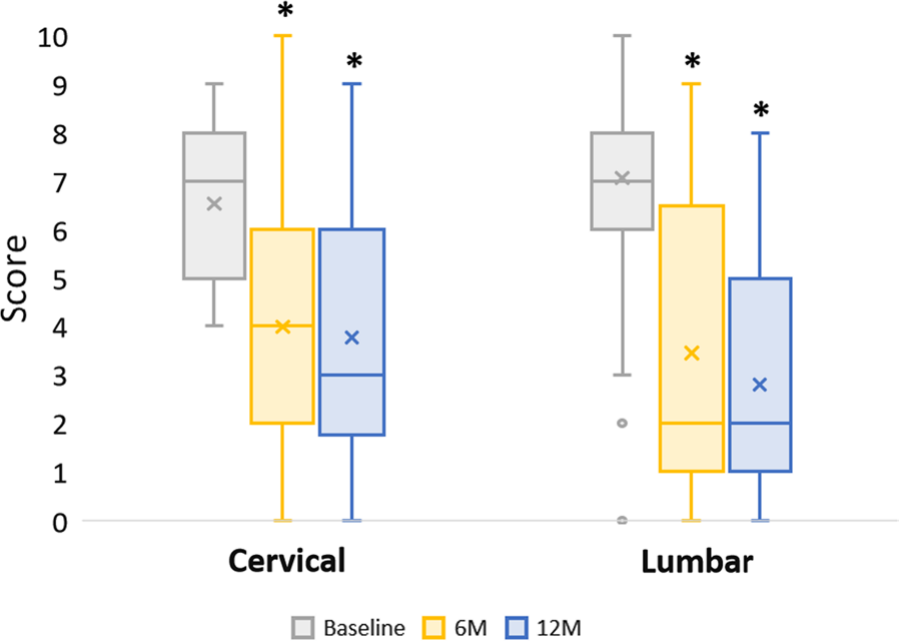
Visual Analog Scale (VAS) Scores. VAS assessment scores presented as Median (IQR, 25th–75th percentile); Cervical baseline: 7 (5–8), 6-month: 4 (2–6), 12-month: 3 (2–6); Lumbar baseline: 7 (6–8), 6-month: 2 (1–6), 12-month: 2 (1–5). * indicates VAS change at each postoperative time point (6-month and 12-month) was significantly improved compared to the baseline score (*p* < 0.001)

**Fig. 2:**
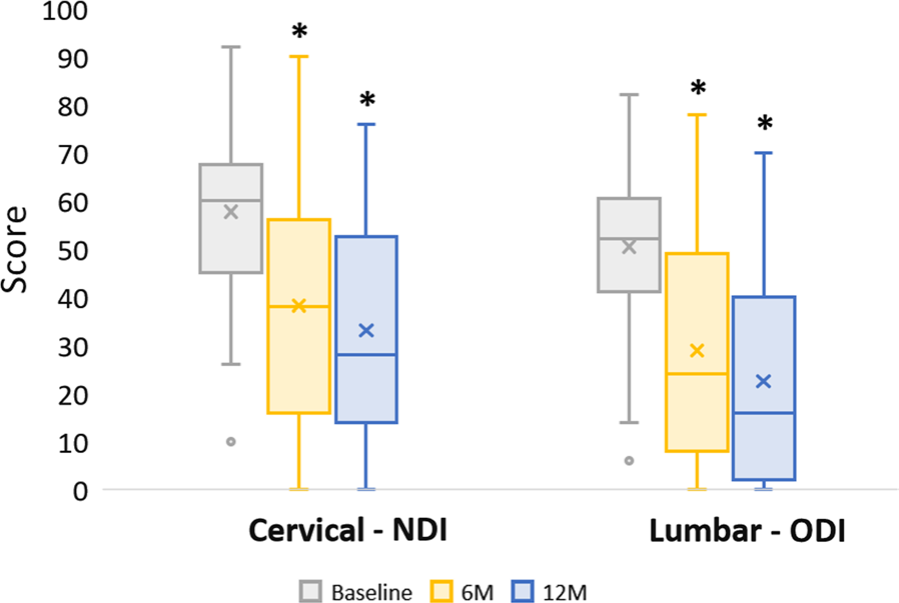
Neck Disability Index (NDI) and Oswestry Disability Index (ODI) Scores. NDI and ODI scores presented as Median (IQR, 25th–75th percentile); Cervical NDI baseline: 60 (47–67), 6-month: 38 (16–56), 12-month: 28 (15–50); Lumbar ODI baseline: 52 (43–60), 6-month: 24 (10–48), 12-month: 16 (2–38). * indicates NDI and ODI change at each postoperative time point (6-month and 12-month) was significantly improved compared to the baseline score (*p* < 0.001)

All patient-reported outcomes demonstrated statistically significant improvements (*p* < 0.001) in pain and function at 6 and 12 months as compared to baseline for VAS-pain, NDI, and ODI (Table 4, Fig. 1, Fig. 2). The greatest improvement in pain and disability scores from baseline were observed at the 12-month evaluation. All patients (100%) reached the minimum clinically significant mean reduction in VAS-pain (− 2.6 cervical, − 2 lumbar), NDI (− 17.3%), and ODI (− 10%) at 12 months [12, 13]. The mean change in VAS-pain scores at 6 and 12 months were − 3.1 (− 2.5 cervical, − 3.7 lumbar) and − 3.5 (− 2.7 cervical, − 4.3 lumbar), respectfully. At 6 and 12 months, the mean change in NDI percentage points were − 19.5% and − 24.6%, respectively, and mean change in ODI percentage points were − 21.4% and − 27.9%, respectively.

**Table 4:** Pain and Disability Scores

	Baseline	6 months	12 months	Baseline vs. 6mo (*p*-value)	Baseline vs. 12mo (*p*-value)
Pain VAS					
(Mean score ± SD)					
Cervical	6.5 ± 1.0	4.0 ± 2.6	3.8 ± 2.6	< 0.001	< 0.001
Lumbar	7.1 ± 1.0	3.4 ± 3.0	2.8 ± 2.5	< 0.001	< 0.001
Disability Index					
(% Mean score ± SD)					
Cervical—NDI	57.7 ± 16.8	38.2 ± 23.0	33.1 ± 21.8	< 0.001	< 0.001
Lumbar—ODI	50.4 ± 16.6	29.0 ± 23.3	22.5 ± 22.2	< 0.001	< 0.001

Overall, there was a 51.5% improvement in VAS-pain (41.5% cervical, 60.6% lumbar), 42.6% in NDI, and 55.4% in ODI from baseline to 12 months. Comparison of subjective measures between 6- and 12-month postoperative follow-up were not statistically significant.

### Clinical and radiographic outcomes

#### Per patient

Treating physicians denoted fused or not fused based on their subjective individual evaluation for 42 cervical and 41 lumbar patients. Five patients (2 cervical; 3 lumbar) achieved fusion at 6 months and did not undergo 12-month follow-up. At 12 months, 88.1% (n = 37/42) of cervical and 97.6% (n = 40/41) of lumbar patients achieved fusion (Table 5). Of the patients denoted not fused at 12 months, all were found to have stable constructs with no malalignment of hardware. For the 5 cervical patients: one had been treated for metastatic breast carcinoma prior to surgery, two were suspected to become fully fused within the following 3–6 months, one 3-level construct had moderate adjacent level disc degeneration, and the remaining patient was the only one who underwent revision posterior fusion. For the not fused lumbar patient, the treating physician noted no bridging bone with loosening of sacroiliac (SI) screws, which was an extension of a previous long construct (C2–L5). As expected, the mean time to fusion was significantly lower in cervical than lumber patients (310.2 ± 73.8 vs 349.2 ± 85.9 days respectively, *p* = 0.037).

**Table 5:** Fusion rates

		Bridwell grade (by level), n (%)					
		I	II	III	IV	Successful fusion (I + II)^11^	Fusion by patient (n = 83)
Cervical	6 months (n = 88)	17 (19.3%)	69 (78.4%)	2 (2.3%)	0 (0.0%)	**86 (97.7%)**	
	12 months (n = 65)	39 (60.0%)	25 (38.5%)	1 (1.5%)	0 (0.0%)	**64 (98.5%)**	**37 (88.1%)** (n = 42)
Lumbar	6 months (n = 57)	13 (22.8%)	43 (75.4%)	1 (1.8%)	0 (0.0%)	**56 (98.3%)**	
	12 months (n = 50)	11 (22.0%)	39 (78.0%)	0 (0.0%)	0 (0.0%)	**50 (100.0%)**	**40 (97.6%)** (n = 41)

Bolded numbers indicate overall successful per-level and per-patient fusion rates

#### Per level

**Fig. 3:**
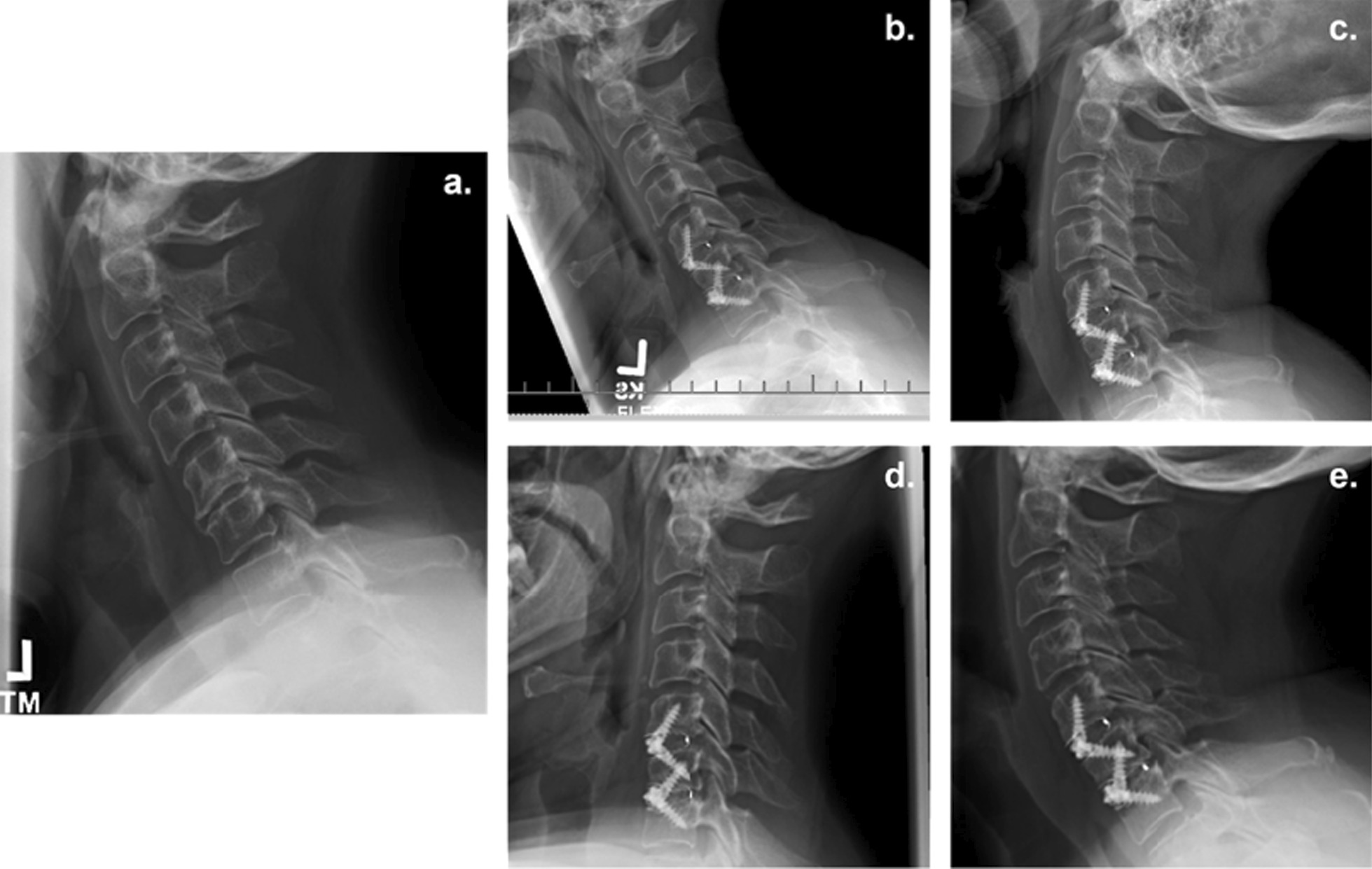
Pre- and post-operative radiographs of a 2-level ACDF using ViBone on a 58-year-old obese, current smoker, type II diabetic female at C5–C6 and C6–C7. **a** Pre-operative lateral radiograph **b** six-month flexion radiograph **c** six-month extension radiograph **d** Twelve-month flexion radiograph **e** Twelve-month extension radiograph

**Fig. 4:**
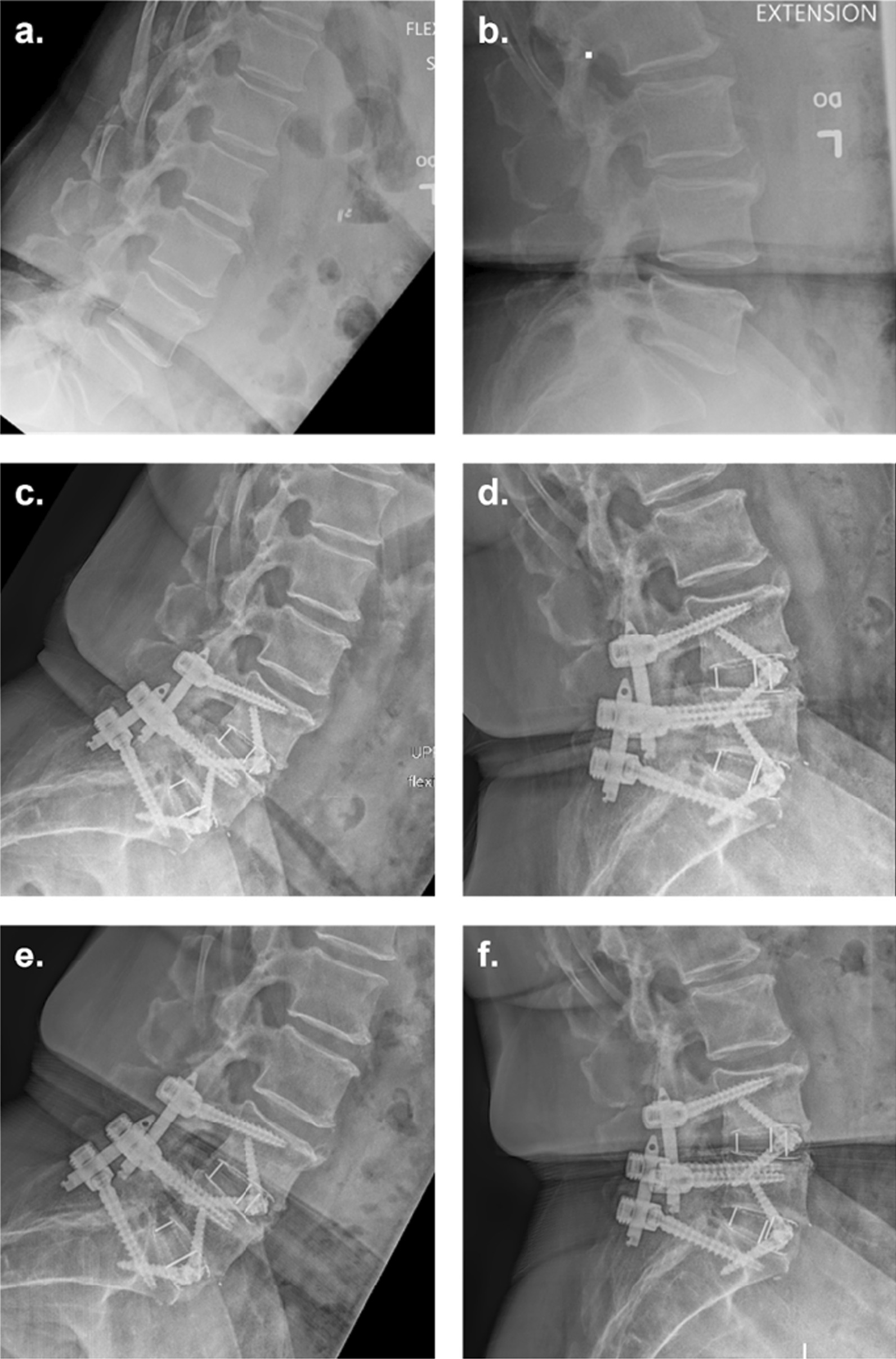
Pre- and post-operative radiographs of a 2-level ALIF using ViBone on a 74-year-old overweight female at L4–L5 and L5–S1. **a** Pre-operative flexion radiograph **b** Pre-operative extension radiograph **c** six-month flexion radiograph **d** six-month extension radiograph **e** Twelve-month flexion radiograph **f** Twelve-month extension radiograph

Independent radiologic evaluation was used to determine per level fusion. A total of 88 cervical levels were reviewed from the available 6-month post-op radiographs and 65 levels were reviewed from the available 12-month post-op radiographs. There were 57 levels reviewed from lumbar radiographs at 6 months post-op and 50 levels at 12 months post-op. Based on the Bridwell interbody fusion grading system (Table 1) [10] and literature definitions for successful fusion (Grade I and II) [11], 97.7% (n = 86/88) of cervical levels demonstrated successful fusion at 6 months and 98.5% (n = 64/65) were fused at 12 months (Table 5). The one cervical level that did not achieve fusion at 12 months was from an obese and hypertensive female patient who underwent a 3-level fusion at C4-7. Among patients who underwent lumbar fusion, 98.3% (n = 56/57) of levels achieved successful fusion at 6 months and 100% (n = 50/50) were fused at 12 months (Table 5). Example radiographs displaying baseline and Grade I successful fusion at 6- and 12-month follow-up for cervical and lumbar patients are provided in Figs. 3 and 4, respectively.

### Safety

There were no graft or procedure-related adverse events reported.

## Discussion

Spinal fusion is a common procedure used to treat many conditions causing spinal instability. ICBG has been the gold standard graft for spinal fusion because it possesses osteoconductive, osteoinductive, and osteogenic qualities that are necessary for bone arthrodesis. However, ICBG harvest is associated with a second operative site, pain, and morbidity [3]. Thus, several surgical techniques and graft materials are now available to improve clinical outcomes in spinal arthrodesis. Some bone graft substitutes do not possess all the essential characteristics necessary for successful fusion, and if the fusion graft fails, pseudoarthrosis can lead to poor clinical outcomes or revision surgery [3, 6]. There is a need for bone graft substitutes that increase fusion rates and avoid morbidity of ICBG harvesting.

Recombinant human bone morphogenetic protein-2 (rhBMP-2, Infuse Bone Graft, Medtronic, Memphis, TN) is commonly used and reported to yield high spinal fusion rates without the need for ICBG harvest. However it has also been reported to be associated with significant adverse events from various publications, most notably the Yale University Open Data Access (YODA) Project [14, 15]. Two separate meta-analyses of this data concluded that use of rhBMP-2 is significantly associated with overall adverse events, wound complications, as well as an increased risk of cancer, heterotrophic bone formation, and osteolysis [14, 15]. Thus, there remains a need for bone graft substitutes that yield high fusion rates with low complication risk.

The ability of a bone graft to successfully form new bone (osteogenesis) requires the presence of viable bone-forming cells [16]. Therefore, allografts that contain cells which support bone formation and minimize apoptosis may be clinically beneficial. ViBone VBM contains a comparable number of cells to other commercially available VBMs, yet previous in vitro work demonstrated it had 58% fewer apoptotic cells. This resulted in cell proliferation approximately twice as fast and produced more osteogenic factors compared to control VBM prepared with conventional processing [17]. We are currently unaware of any other similar comparisons of living cell health from one VBM compared to another method of VBM processing. In an athymic nude rat model, ViBone upregulated genes associated with endochondral ossification and continuous bone remodeling [18]. With these promising pre-clinical results, it was necessary to assess the clinical safety and efficacy of this VBM.

The primary aim of this study was to assess the safety and effectiveness of ViBone VBM in cervical and lumbar fusion procedures. To our knowledge, this is the first study reporting results of a VBM used separately in both cervical and lumbar procedures. Solid fusion at 12 months was achieved in 88.1% cervical and 97.6% lumbar patients (98.5% and 100% of levels, respectively). Furthermore, all subjective clinical assessments (VAS-pain, NDI, ODI) exhibited significant improvements at both 6- and 12-month assessments compared to baseline, and all patients reached clinically significant improvements at 12 months. The use of ViBone was not associated with any adverse events or infections and circumvented the need for harvesting ICBG, which highlights the safety of this VBM for spinal fusion procedures.

Our results align with previously published spinal fusion rates for ICBG, and pain/disability scores in spinal fusion studies assessing similar numbers of patients with radiographic imaging and clinician assessments as the main evaluation tools [19–21]. Early studies on spinal fusion with VBM report solid arthrodesis and improved pain and disability scores at 12-month follow up [20, 21]. A review of initial spinal fusion clinical studies concluded that VBM grafts were safe for use in bone grafting since there were no adverse events associated with their use [22]. More recently, two systematic reviews concluded that use of VBMs for spinal fusion led to high fusion and low complication rates [23, 24]. Specifically, 12-month fusion rates reported from using various VBMs in those studies ranged from 88–100% in cervical and 91–99% in lumbar patients, while per-level fusion ranged from 87–93% in cervical and 68–99% in lumbar segments [23, 24]. These findings align well with the results outlined in our study at 12-month evaluation for per-patient (88.1% cervical and 97.6% lumbar) and per-level fusion (98.5% cervical, 100% lumbar), with clinically meaningful improvements in pain and disability scores.

Similar to other studies, our results suggest that VBMs are a promising alternative to ICBG, producing comparable high fusion rates, and no associated adverse events or need for ICBG harvesting. In addition to ViBone, two other commercially available VBMs, Fiber VBM and OsteGro VBM, are also processed by the same manufacturer (Aziyo Biologics, Silver Spring, MD) with a focus on minimized apoptosis, enhanced cell–matrix preservation, and optimized handling characteristics. These minimally processed formulations are designed to improve cellular health and provide higher levels of growth factors to stimulate early and later stage bone formation.

Limitations of this study include the lack of control group(s) and randomization. Also, the variety of surgical techniques and use of other bone graft materials in addition to ViBone may have influenced observed outcomes. Larger studies with longer-term follow-up are necessary to further evaluate the safety and efficacy of ViBone VBM in surgical spine patients.

## Conclusions

The results of this study show positive clinical outcomes and safety of ViBone VBM for cervical and lumbar spinal fusion. Radiographic imaging demonstrates favorable fusion results, similar to previously reported fusion rates of ICBG and other bone graft substitutes. ViBone VBM is a promising bone graft substitute and extender for spinal arthrodesis, which may avoid complications and circumvent the need for harvesting ICBG.

## Data Availability

The dataset generated and analyzed during the current study is available from the corresponding author upon reasonable request.

## Abbreviations

ACDF: Anterior cervical discectomy and fusion
AFIB: Atrial fibrillation
ALIF: Anterior lumbar interbody fusion
BMI: Body mass index
CAD: Coronary artery disease
CBM: Cellular bone matrix
DBM: Demineralized bone matrix
DDD: Degenerative disc disease
HNP: Herniated nucleus pulposus
ICBG: Iliac crest bone graft
IRB: Institutional review board
MSC: Mesenchymal stem cell
MIS: Minimally invasive surgical
NDI: Neck disability index
ODI: Oswestry disability index
PEEK: Polyetheretherketone
PEKK: Polyetherketoneketone
PLIF: Posterior lumbar interbody fusion
SD: Standard deviation
TLIF: Transforaminal lumbar interbody fusion
VAS-pain: Visual analog scale for pain
VBM: Viable bone matrix
